# TLE1 inhibits anoikis and promotes tumorigenicity in human lung cancer cells through ZEB1-mediated E-cadherin repression

**DOI:** 10.18632/oncotarget.19703

**Published:** 2017-07-31

**Authors:** Xin Yao, Tri Pham, Brandi Temple, Selena Gray, Cornita Cannon, Camry Hardy, Kamari Fletcher, Shubha Kale Ireland, Ahamed Hossain, Renwei Chen, Asim B. Abdel-Mageed, Hector Biliran

**Affiliations:** ^1^ Department of Biological and Public Health Sciences, Xavier University of Louisiana, New Orleans, LA 70125, USA; ^2^ Center for Bioengineering, University of California, Santa Barbara, CA 93106, USA; ^3^ Tulane Cancer Center, Tulane University School of Medicine, New Orleans, LA 70112, USA

**Keywords:** TLE1, anoikis, E-cadherin, tumorigenecity, ZEB1

## Abstract

The Transducin-like enhancer of split 1 (TLE1) corepressor protein is overexpressed in human lung tumors and is a putative lung-specific oncogene. However, the molecular mechanism underlying its oncogenic function remains to be delineated. Here, we report an important role of TLE1 in promoting lung tumorigenesis by a mechanism involving induction of anoikis resistance. Using the human lung adenocarcinoma A549 and immortalized bronchial epithelial BEAS-2B cell lines, we observed that TLE1 inhibits anoikis through transcriptional repression of E-cadherin gene. In support of E-cadherin as a downstream target of TLE1 to block anoikis, forced expression of E-cadherin attenuated TLE1-induced anoikis resistance while E-cadherin downregulation decreased the anoikis sensitivity of TLE1 knockdown cells. Furthermore, we determined that E-cadherin expression is transcriptionally induced upon loss of cell attachment and functions as an effector of anoikis. Loss of E-cadherin via the siRNA strategy or exogenous TLE1 expression was sufficient to attenuate anoikis in A549 and BEAS-2B cells. Importantly, we demonstrated that the ZEB1 transcriptional factor is required for TLE1-mediated E-cadherin repression and anoikis resistance. ZEB1 interacted with and recruited the TLE1 to the E-cadherin promoter to impose histone deacetylation and gene silencing. *In vivo*, TLE1 strongly promoted tumorigenicity of A549 cells in a ZEB1-dependent manner. Underscoring its role in anoikis insensitivity of lung cancer cells, the TLE1-mediated E-cadherin repression was negatively regulated by the tumor suppressor Bcl-2 inhibitor of transcription 1 (Bit1) to effect anoikis. These findings identify the ZEB1/TLE1/E-cadherin transcriptional mechanism as a novel pathway that promotes anoikis resistance and oncogenicity of lung cancer cells.

## INTRODUCTION

Transducin-like enhancer of split 1 (TLE1) is a member of the Groucho (Gro)/TLE gene family encoding transcriptional corepressors [1–3]. Like most traditional co-repressors, the Gro/TLE family proteins do not contain a DNA-binding domain and gets recruited to target genes via interaction with known DNA-binding transcriptional factors such as the Hairy-Enhancer of split (HES), Runx, Nkx, and LEF1/Tcf proteins. Although the role of Gro/TLE family proteins as corepressors in several transcriptional pathways including Wnt, Notch, Pax2 and Runx2 has been established, the underlying molecular mechanism(s) for their repressive activity remains to be fully defined. A widely accepted model involves recruitment of chromatin remodeling enzymes such as histone deacetylase (HDAC) to target genes to impose a transcriptionally repressive chromatin structure [4, 5]. Shown via genetic and biochemical studies, the recruitment of HDAC by Gro/TLE proteins results in the removal of acetyl groups from nearby DNA-bound histones leading to a condensed and repressed chromatin state. However, it is likely that TLE1 may depend on other chromatin remodeling proteins to effect transcriptional silencing based on evidence that HDAC inhibitors are unable to completely block the TLE1 repressive function (5). Intriguingly, the Gro/TLE proteins may also associate directly with chromatin via interaction with the amino termini of histone H3 protein as an alternative mechanism to induce transcriptional silencing [6].

As a member of the vertebrate Gro/TLE family of proteins, TLE1 exhibits a well-characterized function in the regulation of nervous system development. In particular, TLE1 exhibits anti-neurogenic activity in mammalian forebrain development. While forced TLE1 expression in transgenic mice inhibits neuronal development in the forebrain *in vivo* [7], ectopic TLE1 expression in neural progenitor cells in culture promoted their un-differentiation status with concomitant increased proliferative ability [8]. In addition to its role as an anti-differentiation factor in neurogenesis, TLE1 exhibits a pro-survival and anti-apoptotic function in several mammalian cellular models. Forced expression of TLE1 induced anchorage-independent survival and growth of chicken embryo fibroblast cells [9]. TLE1 in conjunction with Forkhead box protein G1 (FoxG1) promoted survival in post-mitotic neurons [10]. The pro-survival function of TLE1 has also been observed in malignant cells, particularly in synovial sarcoma cells [11] and breast cancer cells [12].

In light of its anti-differentiation and growth promoting function in cellular systems, it is not surprising that TLE1 has been implicated in the pathogenesis of cancer. First, TLE1 is aberrantly expressed or upregulated in various types of human cancer including synovial sarcoma [11], breast [12] and lung cancer [13]. Second, in line with the notion of TLE1 as an oncogenic factor, TLE1 is highly expressed in proliferative epithelial tissues as well as in diseased metaplastic and neoplastic transformed states [14]. Perhaps, the most convincing evidence is from the transgenic mice overexpressing the mouse homolog Grg1, which exhibited lung tumors resembling human lung adenocarcinoma [13]. This latter data suggests TLE1 as a putative lung-specific oncogene. Although the survival signaling ErbB1 and ErbB2 signaling pathways have been shown to be activated in Grg1-induced lung adenocarcinomas, the molecular mechanism underlying the TLE1-induced lung oncogenicity remains to be fully elucidated.

Recently, we have uncovered a novel function of the TLE1 corepressor as an effector of EMT in lung cancer cells through transcriptional silencing of the epithelial marker E-cadherin [15]. Based on numerous studies indicating that an EMT phenotype and particularly the loss of E-cadherin expression is associated with cell survival [16, 17], we investigated here the role of TLE1 as an effector of anoikis resistance in lung cancer cells. Here, we show that the E-cadherin expression is transcriptionally induced upon loss of cell attachment, and upregulated E-cadherin expression enhances anoikis in lung cancer cells. Direct transcriptional suppression of E-cadherin expression by TLE1 via the transcription factor ZEB1 conferred enhanced anoikis insensitivity, anchorage-independent growth *in vitro*, and tumorigenicity *in vivo* of lung cancer cells. As a critical molecular event underlying lung cancer cell anoikis resistance, the TLE1-mediated repression of E-cadherin acted as a downstream target of the anoikis function of the tumor suppressor Bcl-2 inhibitor of transcription 1 (Bit1) [18, 19]. Our collective results identify the ZEB1/TLE1 as a novel transcriptional mechanism in regulating E-cadherin expression and lung oncogenicity.

## RESULTS

### E-cadherin expression is induced following cell detachment and promotes anoikis in A549 and BEAS-2B cells

**Figure 1:**
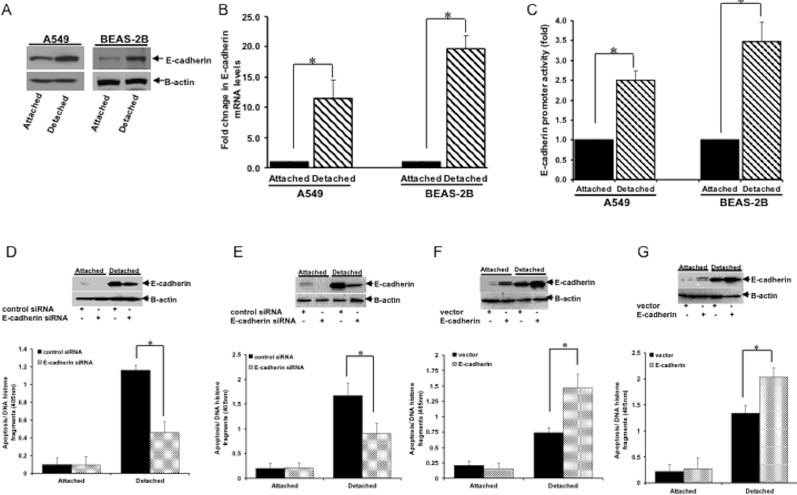
Induction of E-cadherin expression upon loss of cell attachment induces anoikis **(A)** A549 and BEAS-2B cells were cultured in normal culture condition (attached) or in suspension (detached) as described in Materials and Methods, and then harvested and subjected to immunoblotting with the indicated antibodies. **(B)** Cells cultured in attached and detached conditions were subjected to real-time PCR to assess the E-cadherin mRNA level. **(C)** Attached and detached cells were subjected to luciferase promoter assay to determine the E-cadherin promoter activity. **(D** and **E)** A549 (D) and BEAS-2B (E) cells transfected with control- or E-cadherin specific siRNAs were cultured in attached or detached conditions (as described in Materials and Methods), and then cells were harvested and subjected to immunoblotting with the indicated antibodies (upper panel) or cell death ELISA (lower panel). **(F** and **G)** A549 (F) and BEAS-2B (G) cells transfected with the empty vector or E-cadherin construct were cultured in attached or detached conditions and then subjected to immunoblotting (upper panel) or cell death ELISA (lower panel). In B to G, three independent experiments were performed and statistical significance was assessed by Student's t-test (*p < 0.05). Error bars displayed on graphs represent mean ± S.D.

Loss of E-cadherin expression has been associated with induction of anoikis resistance in mammary tumor cells [16, 17]. To address the role of E-cadherin in the anoikis sensitivity of lung cancer cells, we first examined if E-cadherin expression at the protein level is regulated by loss of cell attachment. As shown in Figure 1A, loss of cell attachment triggered an increase in the steady-state level of E-cadherin protein in human adenocarcinoma A549 cells. Indeed, detached cells exhibited increased plasma membrane localization of E-cadherin as compared to attached cells (Supplementary Figure 1). The increased E-cadherin protein levels in detached cells are associated with an increase in E-cadherin mRNA level (Figure 1B) and E-cadherin promoter activity (Figure 1C), indicating that loss of cell attachment triggered transcriptional induction of E-cadherin expression. To complement these findings, we also examined the E-cadherin protein and mRNA expression levels and the E-cadherin reporter activity in the immortalized human bronchial epithelial BEAS-2B cell line following detachment. Loss of cell attachment in these cells similarly showed an increase in the E-cadherin protein levels (Figure 1A, Supplementary Figure 1) with concomitant upregulation of the E-cadherin mRNA transcript (Figure 1B) and reporter activity (Figure 1C). Together, these findings suggest that loss of cell attachment triggered transcriptional induction of E-cadherin expression in lung cancer cells.

To directly assess the functional role of E-cadherin in anoikis in lung cancer cells, the A549 cells were treated with control or E-cadherin siRNA pool and then cultured in suspension. As shown in Figure 1D, forced downregulation of the E-cadherin expression attenuated anoikis. Acute knockdown of E-cadherin similarly decreased the anoikis sensitivity of BEAS-2B cells (Figure 1E). To complement these findings, we investigated the effect of ectopic E-cadherin expression on anoikis induction in A549 and BEAS-2B cells. Indeed, exogenous E-cadherin potentiated the level of apoptosis in detached A549 (Figure 1F) and BEAS-2B cells (Figure 1G). Importantly, the highly anoikis resistant lung cancer H460 cells, which exhibit undetectable levels of E-cadherin protein, were induced to undergo detachment-induced apoptosis upon exogenous expression of E-cadherin (Supplementary Figure 2). Taken together, these data suggest that expression of E-cadherin is transcriptionally induced following the loss of cell attachment and promotes anoikis sensitization of lung cancer cells.

### TLE1 enhances anoikis resistance through E-cadherin repression

**Figure 2:**
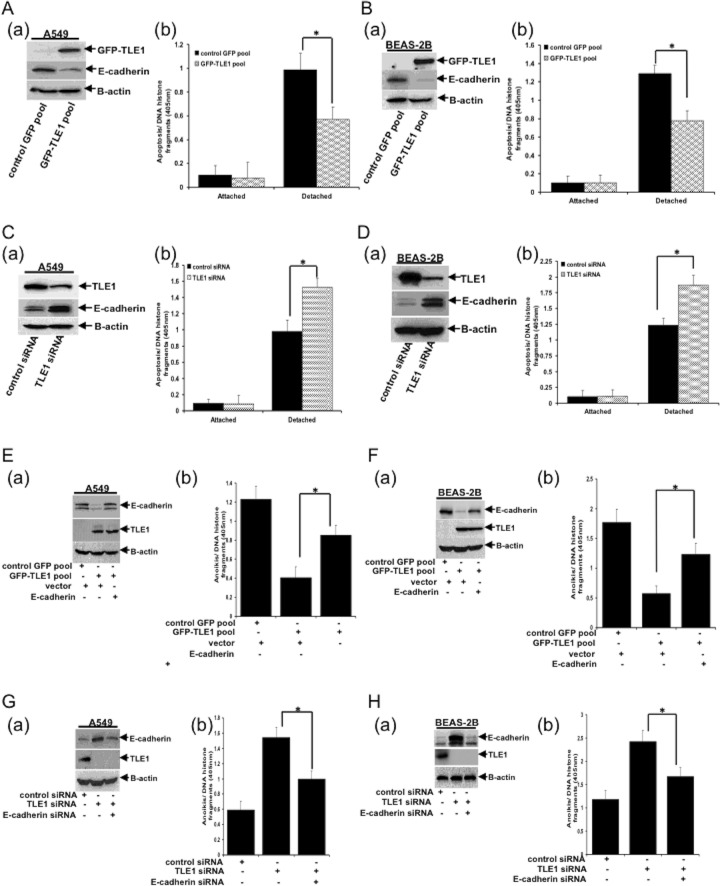
TLE1 induces anoikis resistance through silencing of E-cadherin expression **(A)** (a) Stable control GFP and GFP-TLE1 A549 cell pools were subjected to immunoblotting with anti-GFP (to confirm the expression of exogenous GFP-TLE1) and anti-E-cadherin antibodies. (b) Control GFP and GFP-TLE1 cells were cultured in attached or detached conditions for 24 h followed by cell death ELISA. **(B)** (a) Stable control GFP and GFP-TLE1 BEAS-2B cell pools were subjected to immunoblotting with anti-GFP and anti-E-cadherin antibodies. (b) Control GFP and GFP-TLE1 cells were cultured in attached or detached conditions for 24h followed by cell death ELISA. **(C** and **D)** (a) A549 (C) and BEAS-2B (D) cells were transfected with control- or TLE1 siRNA pools, and 24h later cells were subjected to immunoblotting with the indicated antibodies. (b) Control siRNA and TLE1 siRNA-treated A549 (C) and BEAS-2B (D) cells were cultured in attached or detached conditions for 24h followed by cell death ELISA. **(E** and **F)** (a) Control and GFP-TLE1 expressing A549 (E) and BEAS-2B (F) cells were transfected with vector or E-cadherin construct as indicated and then subjected to immunoblotting with the indicated antibodies. (b) Control and GFP-TLE1 A549 (E) and BEAS-2B (F) cells transfected with vector or E-cadherin construct were cultured in detached condition for 24h followed by cell death ELISA assay. **(G** and **H)** (a) A549 (G) and BEAS-2B (H) cells were treated with the indicated siRNAs as described in materials and methods and then subjected to immunoblotting. (b) siRNA treated cells were cultured in detached condition for 24h followed by cell death ELISA. In A to H, results are representative of three independent experiments and statistical significance was assessed by Student's t-test (*p < 0.05). Error bars represent mean ± S.D.

The TLE1 corepressor has been previously found to promote EMT by transcriptionally repressing E-cadherin expression in lung cancer cells [15]. Considering that anoikis resistance is a hallmark of EMT [20] and that loss of E-cadherin expression circumvents anoikis [16, 17], we investigated the role of TLE1 in the anoikis induction in lung cancer cells. First, we examined the effect of exogenous TLE1 on anoikis sensitivity of A549 cells (Figure 2A). Consistent with our previous results [15], exogenous TLE1 expression repressed E-cadherin expression (Figure 2A, panel a) and significantly decreased the level of apoptosis in detached A549 cells (Figure 2A, panel b). It is noteworthy that the control and TLE1 expressing cells had the same level of spontaneous apoptosis when grown attached to a culture dish. In BEAS-2B cells (Figure 2B), ectopic TLE1 significantly suppressed E-cadherin expression (panel a) and attenuated the anoikis induction (panel b). To validate these findings, we then examined the effect of downregulating endogenous TLE1 expression on anoikis sensitivity of the A549 and BEAS-2B cell lines. As shown in Figure 2C, acute knockdown of endogenous TLE1 expression in A549 cells via the TLE1 specific siRNA pool induced E-cadherin expression (panel a) and enhanced the level of detachment-induced apoptosis (panel b). Downregulating endogenous TLE1 expression had no effect on basal apoptosis in attached cells. Similar results were observed following knockdown of endogenous TLE1 expression in BEAS-2B (Figure 2D). These collective findings suggest that TLE1 represses E-cadherin expression and promotes anoikis resistance in lung cancer cells.

To determine whether loss of E-cadherin directly contributes to TLE1-mediated anoikis resistance, we tested whether forced expression of E-cadherin in TLE1 expressing cells would restore their anoikis sensitivity. As shown in Figure 2E, ectopic E-cadherin expression increased the level of detachment-induced apoptosis in TLE1 expressing A549 cells. Similarly, forced expression of E-cadherin in TLE1-expressing BEAS-2B cells reversed the anoikis resistance imposed by exogenous TLE1 (Figure 2F). To complement these findings, we determined the effect of downregulating E-cadherin expression on anoikis sensitivity of TLE1 knockdown A549 (Figure 2G) and BEAS-2B cells (Figure 2H). Forced knockdown of E-cadherin expression attenuated the enhanced anoikis sensitivity imposed by the loss of endogenous TLE1. These data suggest that E-cadherin repression is necessary for TLE1 to induce anoikis resistance in lung cancer cells.

### ZEB1 is required for TLE1-induced E-cadherin repression and anoikis resistance

**Figure 3:**
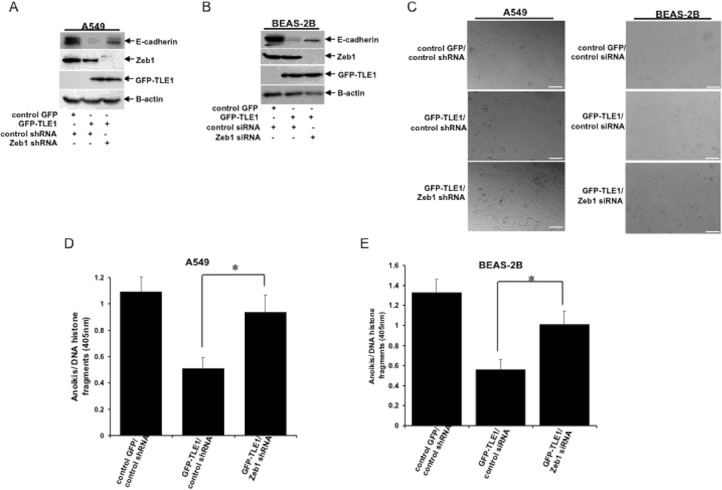
Knockdown of ZEB1 attenuated the TLE1-induced E-cadherin repression and anoikis resistance **(A)** Stable control GFP/control shRNA, GFP-TLE1/control shRNA, and GFP-TLE1/ZEB1 shRNA A549 cell pools were generated as described in Materials and Methods and were subjected to immunoblotting with the indicated antibodies. **(B)** Stable control GFP and GFP-TLE1 BEAS-2B cells were transfected with control or ZEB1 siRNA pools, and 24h later cells were subjected to immunoblotting with the indicated antibodies. **(C)** Control GFP/control shRNA, GFP-TLE1/control shRNA, and GFP-TLE1/ZEB1 shRNA A549 cells (left panel) and control GFP and GFP-TLE1 BEAS-2B cells treated with control or ZEB1 siRNAs (right panel) were subjected to bright field microscopy (10x magnification) under normal culture conditions and representative photographs are shown; scale bar, 50 μm. **(D)** Stable control GFP/control shRNA, GFP-TLE1/control shRNA, and GFP-TLE1/ZEB1 shRNA A549 cells were cultured in suspension for 24h and then subjected to cell death ELISA assay. **(E)** Control GFP and GFP-TLE1 BEAS-2B cells treated with control or ZEB1 siRNAs were cultured in suspension for 24h and then subjected to cell death ELISA assay. In D and E, three independent experiments were performed in triplicates, * indicates p<0.05 by Student's t test. Error bars represent mean ± S.D.

As a corepressor, TLE1 lacks a DNA binding motif and relies on transcription factor to get recruited to target genes. Among the well-known EMT promoting transcription factors, ZEB1 has been uniquely correlated with loss of E-cadherin expression [21] and anchorage-independent growth [22] in human lung cancer cell lines. Consistent with our previous results [19], knockdown of endogenous ZEB1 expression inhibited the E-cadherin repression induced by TLE1 in A549 cells (Figure 3A, Supplementary Figure 3). Similar abrogation of the TLE1-mediated E-cadherin repression was observed in BEAS-2B cells following knockdown of ZEB1 expression (Figure 3B, Supplementary Figure 3). In line with the induction of the epithelial marker E-cadherin expression following loss of ZEB1 expression in TLE1 expressing cells, the elongated, flattened spindle-shaped morphology of TLE1 expressing A549 (left panel) and BEAS-2B (right panel) cells was transformed into a more compact epithelial phenotype upon depletion of ZEB1 (Figure 3C). These findings suggest that the ability of TLE1 to repress E-cadherin expression depends, at least in part, on ZEB1.

The effect of ZEB1 depletion on TLE1-mediated anoikis resistance was then examined. Consistent with our earlier findings (Figure 2A-2D), exogenous TLE1 expression decreased the level of detachment-induced apoptosis in both A549 (Figure 3D) and BEAS-2B (Figure 3E) cells. Importantly, the specific knockdown of ZEB1 expression attenuated the anoikis resistance in TLE1 expressing cells (Figure 3D and 3E). Taken together, these results suggest that ZEB1 is required for TLE1 to mediate E-cadherin repression and anoikis resistance.

### ZEB1 recruits the TLE1 corepressor to repress E-cadherin transcription

**Figure 4:**
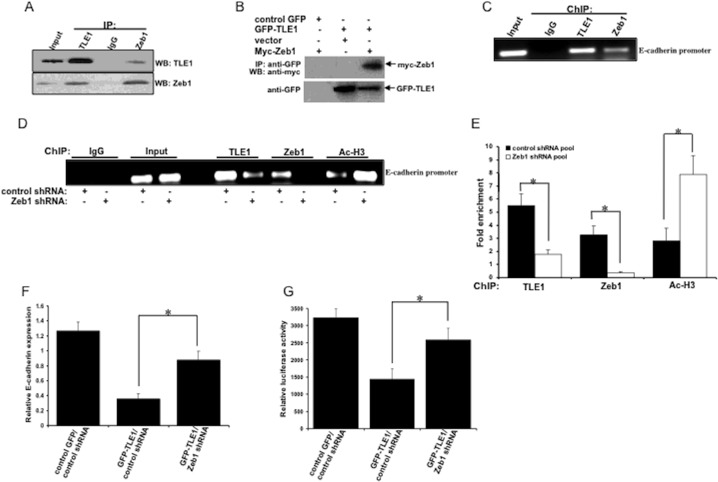
TLE1 is recruited to the E-cadherin promoter and represses E-cadherin transcription in a ZEB1-dependent manner **(A)** A549 cell extracts were prepared and immunoprecipitated with antibodies with TLE1, ZEB1, and control IgG, and the resulting immunoprecipitate complexes were subjected to immunoblotting with the indicated antibodies. **(B)** Control GFP and GFP-TLE1 A549 cells were transfected with vector or Myc-tagged ZEB1 construct as indicated, and 24 h later cells were harvested and cell extracts were prepared, immunoprecipitated with agarose-immobilized anti-GFP, and immunoblotted with anti-GFP and anti-Myc antibodies. **(C)** A549 cells were analyzed by ChIP assay. Chromatin was precipitated using ChIP-validated antibodies against TLE1, ZEB1, and control IgG as detailed in the materials and methods. The E-cadherin promoter sequence was amplified by PCR and subjected to agarose gel electrophoresis. The ChIP experiments were repeated at least three times and a representative experiment is shown. **(D** and **E)** Stable control and ZEB1 shRNA A549 cell pools were subjected to ChIP assay with ChIP-validated antibodies against TLE1, ZEB1, acetyl-histone H3 (Ac-H3), and control IgG. The E-cadherin promoter fragment was amplified by PCR and subjected to agarose gel electrophoresis. The ChIP experiments were repeated at least three times and a representative experiment is shown in D. Enrichment of the E-cadherin promoter fragment in TLE1-ChIP, ZEB1-ChIP, and acetyl-histone 3-ChIP over IgG-antibody is shown in E. **(F** and **G)** Stable control GFP/control shRNA, GFP-TLE1/control shRNA, and GFP-TLE1/ZEB1 shRNA A549 cells were subjected to real-time PCR analysis using specific E-cadherin primers (F) to assess for E-cadherin mRNA levels and E-cadherin promoter luciferase activity assay (G) to quantify the E-cadherin promoter activity. In E, F, and G, three independent experiments were performed in triplicates, * indicates p<0.05 by Student's t test. Error bars represent mean ± S.D.

As an EMT-promoting transcription factor, ZEB1 recruits corepressors such as CtBP to repress target gene expression including that of E-cadherin [23]. Given that ZEB1 has been shown to be the key transcription factor in repressing E-cadherin expression in lung cancer cells [21, 22], we then examined if ZEB1 functions as the DNA-binding transcription factor mediating the observed TLE1-mediated E-cadherin repression. First, we determined if ZEB1 interacts with TLE1 in A549 cells. As shown in Figure 4A, immunoprecipitation with anti-TLE1 and blotting of the precipitate with anti-ZEB1 antibodies revealed ZEB1/TLE1 complexes. Conversely, immunoprecipitation with anti-ZEB1 and subsequent blotting of the precipitate with anti-TLE1 also showed ZEB1/TLE1 complexes. Similar interaction of exogenous TLE1 with ectopic ZEB1 was observed in A549 cells (Figure 4B). We then analyzed whether the ZEB1/TLE1 complex could be assembled on the E-cadherin promoter via chromatin immunoprecipitation assays. As shown in Figure 4C, the ZEB1 and TLE1 proteins bind to the E-cadherin promoter. Importantly, knocking down ZEB1 expression via the specific ZEB1 shRNA not only blocked the binding of ZEB1 but also that of TLE1 (Figure 4D and 4E). We have previously shown that the TLE1 corepressor recruits HDAC to impose histone deacetylation on the E-cadherin promoter [15]. Consistently, abrogation of TLE1 binding on the E-cadherin promoter by ZEB1 depletion was associated with an increase in histone acetylation (Figure 4D and 4E). These findings suggest that the transcription factor ZEB1 recruits TLE1 to the E-cadherin promoter.

The impact of the ZEB1/TLE1 complex formation on E-cadherin transcription was then investigated. Knocking down ZEB1 expression in TLE1 expressing A549 cells resulted in increased level of E-cadherin mRNA transcript (Figure 4F). Consistently, ZEB1 depletion abrogated the repressed E-cadherin promoter activity in TLE1 expressing cells (Figure 4G). These collective findings suggest that ZEB1 recruits TLE1 to the E-cadherin promoter to repress transcription.

### TLE1 induces anchorage-independent growth *in vitro* and tumorigenicity *in vivo* in a ZEB1 dependent manner

**Figure 5:**
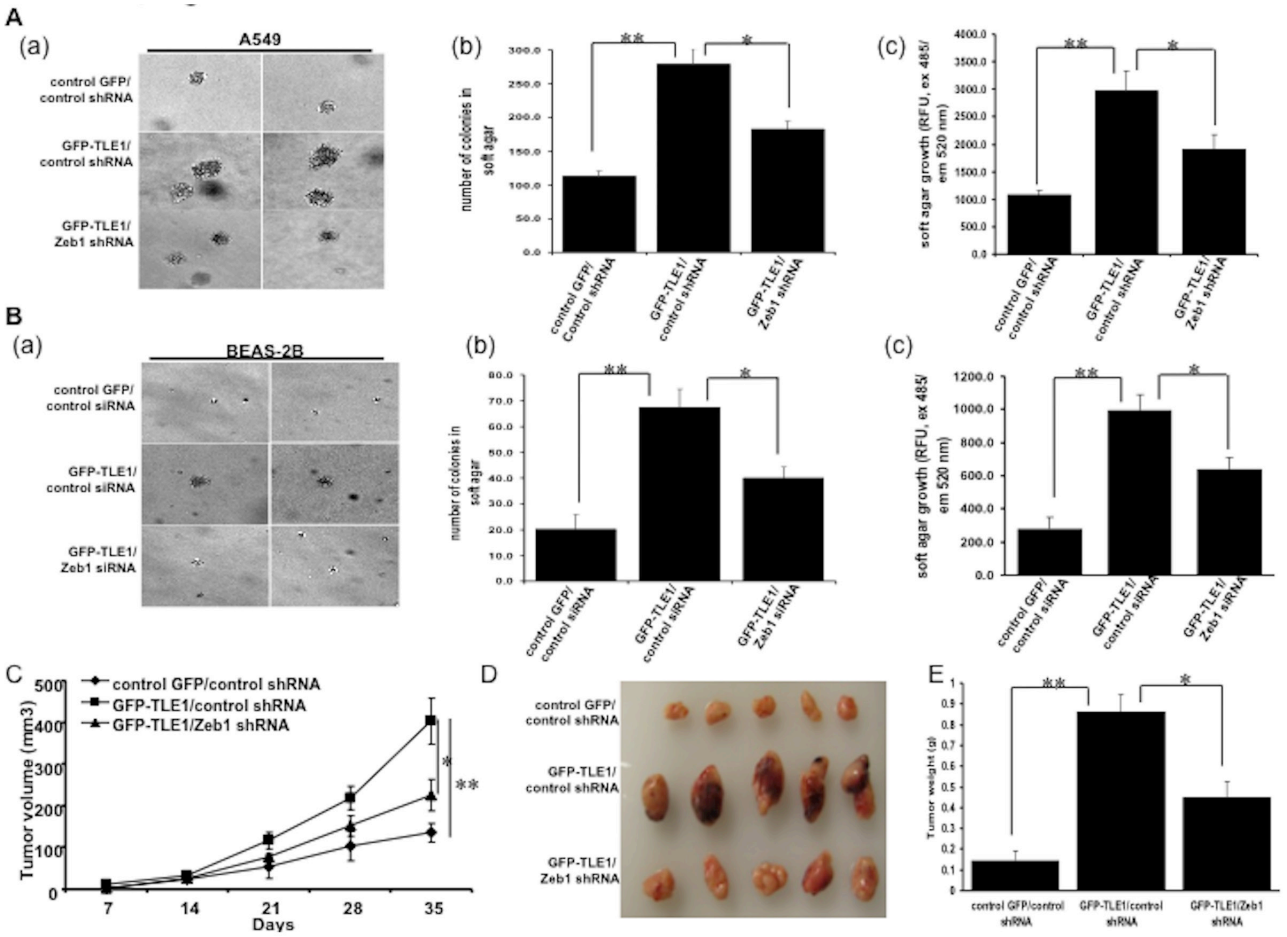
TLE1 promotes anchorage-independent growth *in vitro* and tumorigenicity *in vivo* in a ZEB1 dependent manner **(A** and **B)** (a) Stable control GFP/control shRNA, GFP-TLE1/control shRNA, and GFP-TLE1/ZEB1 shRNA A549 cells (A) and control GFP and GFP-TLE1 BEAS-2B cells treated with control or ZEB1 siRNAs (B) were subjected to a soft agar assay as described in Materials and Methods and the representative colonies are shown. (b) and (c) The extent of colony formation by the cells was quantified by counting the number of visible colonies with a diameter greater than 30 uM (b) and by alamar fluorescence assay (c). **(C, D** and **E)** Stable control GFP/control shRNA, GFP-TLE1/control shRNA, and GFP-TLE1/ZEB1 A549 cells were injected subcutaneously in 8-week old BALB/c nude mice (n = 5/group). Tumors were measured periodically with a caliper on the days after injection. Shown are the mean tumor volumes (±SD) as indicated in C. Mice were then anesthetized and sacrificed on day 35 after injection. Subcutaneous tumors were surgically excised, photographed (D), and weighed wherein the approximate mean tumor weight (±SD) is indicated in (E). In A, B, C, and E, * indicates p<0.05, ** indicates p<0.01 by Student's t test.

Since anoikis resistance is an essential requirement for anchorage-independent growth, a parameter associated with malignant transformation [20], we then examined whether TLE1 can drive the anchorage-independent growth of lung cancer cells. As shown in Figure 5A (panels a and b), exogenous TLE1 expression enhanced the anchorage-independent growth potential of A549 cells, as evidenced by the increase in the number and size of colonies in soft agar. The enhanced growth of TLE expressing cells in soft agar was further quantified by alamar blue staining (Figure 5A, panel c). This observed increased anchorage-independent growth of TLE1 expressing A549 cells was significantly attenuated by the concurrent downregulation of ZEB1 expression. Intriguingly, the minimal colony forming ability of the immortalized human bronchial epithelial BEAS-2B cells in soft agar was significantly enhanced by ectopic TLE1, and such TLE1-mediated increase in anchorage-independent growth was also dependent on ZEB1 (Figure 5B, panels a-c). It is noteworthy that exogenous TLE1 expression inhibited the anchorage-dependent growth of A549 ([15], Supplementary Figure 4) and BEAS-2B (Supplementary Figure 4) cells. Hence, the observed enhanced anchorage-independent growth in soft agar is likely due to inhibition of anoikis. These collective results suggest that TLE1 is an effector of anchorage-independent growth of lung cancer cells, at least in part, through the transcription factor ZEB1.

To directly assess the impact of TLE1 on lung cancer growth *in vivo*, the control/control shRNA, TLE1/control shRNA, and TLE1/ZEB1shRNA A549 cells were injected subcutaneously into nude mice. As shown in Figure 5C and 5D, the TLE1/control shRNA expressing cells showed increased primary tumor growth kinetics as compared to control/control shRNA cells. The average weight of TLE1 expressing tumors was approximately 3.5-fold heavier than the control tumors (Figure 5E). Importantly, knockdown of ZEB1 significantly attenuated the enhanced tumorigenicity of TLE1 expressing cells (Figure 5C-5E). These findings suggest that TLE1 expression confers enhanced tumorigenicity of A549 cells in a mouse xenograft model and such tumorigenic function of TLE1 is dependent on ZEB1.

### The tumor suppressor Bit1 promotes anoikis through induction of E-cadherin expression by inhibiting TLE1 corepressor function

**Figure 6:**
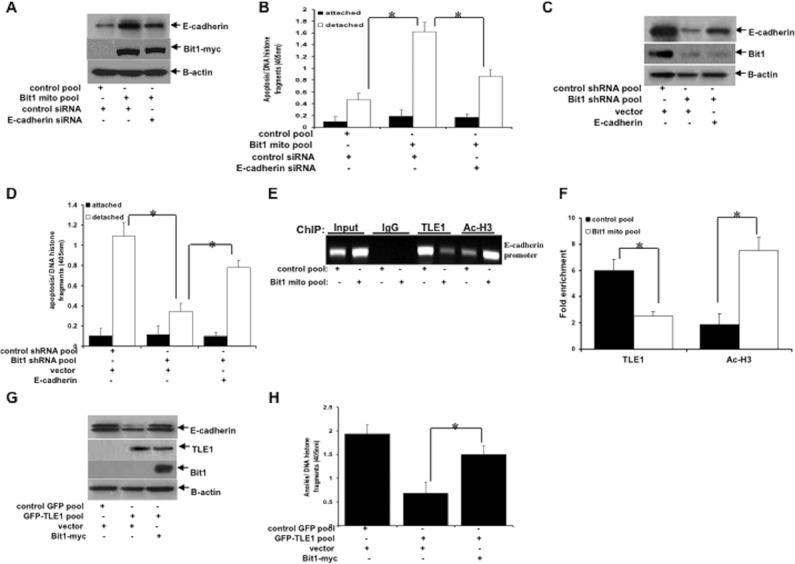
Bit1 induces anoikis through inhibition of the TLE1-mediated transcriptional silencing of E-cadherin expression **(A)** Stable control and Bit1mito pool of A549 cells were transfected with control or E-cadherin siRNA as indicated. 24 h post-transfection, cells were subjected to immunoblotting to detect the expression of Bit1, E-cadherin, and β-actin. **(B)** Stable control and Bit1mito pool of A549 cells transfected with control or E-cadherin siRNA were cultured in attached or detached condition for 48 h followed by cell death ELISA. **(C)** Stable control shRNA and Bit1shRNA pool of A549 cells were transfected with vector or E-cadherin construct as indicated. 24 h post-transfection, cells were harvested and subjected to immunoblotting to detect the expression of Bit1, E-cadherin, and β-actin. **(D)** Stable control shRNA and Bit1shRNA pool of A549 cells transfected with vector or E-cadherin construct were cultured in attached or detached condition for 72 h followed by cell death ELISA. **(E** and **F)** Stable control and Bit1mito A549 cells were cultured in suspension for 24h and then subjected to ChIP assay with ChIP-validated antibodies against TLE1, acetyl-histone H3 (Ac-H3), and control IgG. The E-cadherin promoter fragment was amplified by PCR and subjected to agarose gel electrophoresis. The ChIP experiments were repeated at least three times and a representative experiment is shown. Enrichment of the E-cadherin promoter fragment in TLE1-ChIP and acetyl-histone 3-ChIP over IgG-antibody is shown in F. **(G)** Stable control GFP and GFP-TLE1 A549 cells were transfected with vector or Bit1 mito construct as indicated, and 24 h later cells were subjected to immunoblotting with E-cadherin, TLE1, Bit1, and β-actin. **(H)** Stable control GFP and GFP-TLE1 A549 cells transfected with vector or Bit1 mito construct were cultured in suspension for 48h and then subjected to cell death ELISA. In B, D, F, and H, three independent experiments were performed in triplicates, * indicates p<0.05 by Student's t test. Error bars represent mean ± S.D.

We have previously shown that the mitochondrial Bit1 protein exhibits tumor-suppressive function in lung cancer via induction of anoikis [18] and inhibition of EMT [19]. Although the Bit1 inhibition of EMT has been associated with the attenuation of the TLE1-mediated E-cadherin repression, the downstream signaling mechanism underlying Bit1 anoikis function in lung cancer cells remains to be fully delineated. Here, we examined whether E-cadherin is a downstream target of Bit1 in promoting anoikis. Consistent with our previous findings [18, 19], exogenous Bit1 expression induces E-cadherin expression (Figure 6A) and enhances anoikis induction in A549 cells (Figure 6B). Importantly, forced knockdown of E-cadherin expression significantly inhibited the Bit1-mediated anoikis (Figure 6B). Consistent with these results, the enhanced anoikis resistance of Bit1 knockdown A549 cells which was associated with reduced E-cadherin expression was attenuated by ectopic E-cadherin (Figure 6C and 6D). These results suggest that induction of E-cadherin expression is an important molecular event contributing to Bit1 anoikis function.

Based on our findings here that TLE1-mediated transcriptional repression of E-cadherin is a determinant of anoikis resistance, we then investigated the possibility that Bit1 may negatively impact the TLE1 corepressor function on E-cadherin during anoikis. Indeed, exogenous Bit1 expression reduced TLE1 localization with a concomitant increase in acetylated histone on the E-cadherin promoter in detached cells (Figure 6E and 6F). Consistently, exogenous Bit1 expression attenuated the TLE1 induced E-cadherin repression (Figure 6G) and anoikis insensitivity in detached cells (Figure 6H). Taken together, these findings suggest that Bit1 induces anoikis in lung cancer cells through upregulation of E-cadherin expression, at least in part, by inhibiting the TLE1 corepressor function.

## DISCUSSION

We have previously uncovered a novel role of the corepressor TLE1 in promoting cell migration and EMT in lung cancer through transcriptional repression of the epithelial marker E-cadherin [15, 19]. Here, using the human lung adenocarcinoma A549 and immortalized bronchial epithelial BEAS-2B cellular models, we present data in support of TLE1 as an effector of anoikis resistance, which is a determinant of malignant transformation and tumor progression. Our results suggest that TLE1 induces anoikis insensitivity in lung cancer cells by repressing E-cadherin expression, at least in part, via the EMT transcription factor ZEB1. As a downstream target of TLE1, E-cadherin is transcriptionally induced following the loss of cell attachment and functions to promote anoikis. Indeed, knockdown of E-cadherin via siRNA strategy or exogenous TLE1 expression conferred anoikis resistance in these cells. Further, we provide evidence here that the TLE1-mediated repression of E-cadherin is negatively regulated by the tumor suppressor Bit1 to effect anoikis. In line with its function as an effector of anoikis resistance, TLE1 induced anchorage-independent growth *in vitro* and tumorigenicity *in vivo* in a ZEB1-dependent manner.

In addition to its anti-differentiation function during neurogenesis [7, 8], the Groucho TLE1 corepressor also exhibits a pro-survival and anti-apoptotic function in several cellular systems. In chicken embryo fibroblasts, exogenous expression of TLE1 induced significant growth stimulation and conferred anchorage-independent growth [9]. Ectopic TLE1 expression also prevented apoptosis induced by low potassium in neurons [10]. In breast cancer cells, TLE1 confers a survival advantage, at least in part, by blocking the Bit1-mediated apoptosis and anoikis pathway [12]. Based on these observations, it is conceivable that TLE1 may regulate a survival-promoting gene regulatory program by suppressing an apoptosis gene transcription program or alternatively upregulating a survival-promoting gene transcription program. Consistent with the latter possibility, TLE1 positively upregulates the expression of the anti-apoptotic Bcl2 gene [24] and promotes the survival promoting ErbB1 and ErbB2 signaling pathways [13]. In this report, we provide evidence that E-cadherin is a novel target of TLE1 in effecting anoikis resistance and its direct transcriptional repression by TLE1 was sufficient to attenuate anoikis sensitivity of lung cancer cells. In light of the fact that corepressors such as Ctbp may regulate both epithelial and cell survival genes concurrently [25], we cannot exclude the possibility that TLE1 may have additional gene target(s) in blocking anoikis and their identification will be the focus of our future studies.

The epithelial marker E-cadherin gene exhibits tumor-suppressive function, at least in part, by blocking tumor progression, invasion, and metastasis in various tumor models [26]. As an important component of the epithelial adherent junction, E-cadherin is a cell adhesion molecule that keeps epithelial cells tightly bound to each other and its loss of expression results in disaggregation of cancer cells and thereby promoting tumor cell motility and invasiveness. Interestingly, the E-cadherin transmembrane protein aside from mediating cell-to-cell contact also regulates the survival and apoptotic response of epithelial cells upon loss of cell matrix attachment. In particular, knockdown of E-cadherin expression in human breast epithelial cells as well as mouse mammary tumor cells confers anoikis resistance [16, 17]. Consistent with these previous findings, we show here that E-cadherin expression is necessary for anoikis induction in human bronchial epithelial BEAS-2B and human adenocarcinoma A549 cells and its loss of expression confers anoikis resistance. To date, the molecular mechanisms underlying the anoikis function of E-cadherin remains to be fully delineated. It is noteworthy that loss of E-cadherin expression results in activation of numerous transcriptional pathways including the β-catenin/APC/GSK-3/LEF1 pathway, which may account for anoikis resistance in E-cadherin knockdown cells [17]. Suppression of E-cadherin also leads to activation of an Ankyrin G-dependent transcription pathway involving NRAGE/TBX2 repression of the pro-apoptotic tumor suppressor p14ARF [27]. Although the downstream target(s) of E-cadherin in controlling anoikis sensitivity in human bronchial epithelial and lung cancer cells remains to be studied, these collective studies underscore E-cadherin as a multifunctional protein whose downregulation may confer several phenotypic changes including anoikis resistance which is a determinant of tumorigenesis and cancer progression.

The molecular mechanisms underlying the repression of E-cadherin expression has been thoroughly examined with many studies pointing to the important role of transcriptional and epigenetic events [28]. To this end, several DNA-binding transcription factors (EcTRs) have been identified including ZEB1, ZEB2, SNAI1, SNAI2, and Twist 1/2 that bind to the E-boxes within the E-cadherin promoter and repress its transcription. The gene silencing function of EcTRs typically requires binding to corepressor proteins which recruit multiprotein complexes containing repressive chromatin remodeling enzymes such as histone methyltransferase, deacetylases, and polycomb-2 protein. In particular, ZEB1, a key mediator of EMT and E-cadherin suppression in lung cancer cells [21, 22], recruits the traditional corepressor C-terminal-binding protein (CtBP) to the promoter of E-cadherin gene, and the histone modifying complexes bound to CtBP then impose histone repressive marks leading to transcriptional repression [23, 29]. On the other hand, there have been reports demonstrating the existence of CtBP-independent mechanisms of ZEB1 repression of E-cadherin [30, 31]. Consistent with these studies, our findings suggest that the Groucho TLE1 functions as a novel corepressor utilized by ZEB1 in repressing E-cadherin expression in lung cancer cells. It will be interesting to determine if TLE1 functions independently from or synergistically with CtBP to effect full ZEB1-dependent repression of E-cadherin in lung cancer cells. In addition, given that ZEB1 promotes anchorage-independent growth in NSCLC cell lines [22], our findings here raise the possibility that the TLE1-dependent repression of E-cadherin may underlie the ZEB1-dependent cell survival effect.

**Figure 7:**
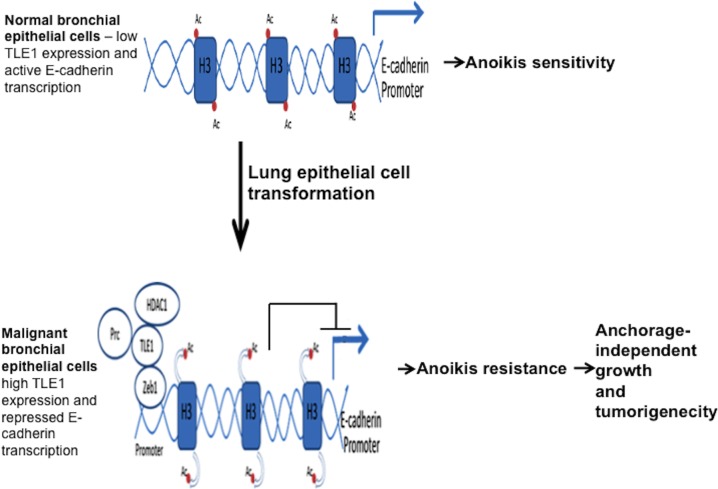
A model depicting the attenuation of E-cadherin expression by TLE1 as a mechanism in promoting anoikis resistance and tumorigenesis in lung cancer In normal bronchial epithelial cells, active E-cadherin transcription and high level of E-cadherin expression maintain their epithelial phenotype and anoikis sensitivity. Upon malignant transformation of lung epithelial cells, E-cadherin expression is downregulated, at least in part, by the ZEB1/TLE1 transcriptional repressor machinery wherein TLE1 and its associated repressive chromatin remodeling enzymes such as Histone deacetylase 1 (HDAC1) are recruited to the E-cadherin promoter to effect histone deacetylation and E-cadherin silencing. The ZEB1/TLE1-mediated transcriptional repression of E-cadherin expression confers anoikis resistance to potentiate the anchorage-independent growth and tumorigenic potential of lung cancer cells. In this model, additional repressive chromatin remodeling proteins such as the histone methylating Polycomb repressive complex (Prc) may also be recruited to the E-cadherin promoter by the ZEB1/TLE1 repressor complex.

Among the Groucho transcriptional corepressor proteins, TLE1 has been previously demonstrated to be a putative lung-specific oncogene [13]. Transgenic mice overexpressing Grg1, a mouse homolog of TLE1, exhibited lung tumors which resemble human lung adenocarcinomas. However, the mechanism underlying TLE1 lung oncogenicity is yet to be examined. Here, we propose a model wherein TLE1 promotes tumorigenicity of lung cancer cells via induction of anoikis resistance through attenuation of the expression of the tumor suppressor gene E-cadherin (Figure 7). This model is consistent with the notion that anoikis resistance is a prerequisite for tumor growth *in vivo* as previously documented [32]. Lacking a DNA binding domain, TLE1 in association with chromatin modifying enzymes such as the Histone deacetylase 1 (HDAC1) is recruited to the E-cadherin promoter via the transcription factor ZEB1 to effect transcriptional repression. Having a dual role in potentiating EMT [15] and anoikis resistance as described here, the ZEB1/TLE1-mediated transcriptional silencing of the tumor suppressive E-cadherin expression represents an important molecular event that may impact the initiation and progression of lung malignancy. As such, the TLE1/E-cadherin nuclear pathway is negatively regulated by the tumor suppressor Bit1 to induce anoikis and inhibit EMT [18, 19]. The exact mechanisms underlying the negative regulation of TLE1 corepressor function by Bit1 remains the subject of active investigation in our laboratory.

In summary, the findings presented here have identified the ZEB1/TLE1 as a new transcriptional mechanism in the suppression of E-cadherin in lung cancer cells. The ZEB1/TLE1-mediated suppression of E-cadherin expression may represent a new pathway utilized by lung cancer cells to acquire anoikis resistance and anchorage-independent growth *in vitro*, and tumorigenicity *in vivo*. Hence, specific inhibition of the ZEB1/TLE1 nuclear pathway is a viable therapeutic strategy to circumvent lung cancer disease initiation and progression.

## MATERIALS AND METHODS

### Cell culture and transfection assays

The human lung adenocarcinoma cell line A549 from American Type Culture Collection (ATCC) was cultured in Dulbecco's modified Eagle's medium (DMEM) with glutamine containing 10% fetal bovine serum, penicillin, and streptoMycin. The stable A549 control GFP and GFP-TLE1 pool of cells were generated by transduction with the lentiviral GFP-TLE1 or the empty control GFP construct (Open Biosystems, Huntsville, AL) as described previously [15] and cultured in the 20μg/ml Blasticidin S (Invitrogen, Carlsbad, CA). To generate the stable A549 derived control GFP/control shRNA, GFP-TLE1/control shRNA, and GFP-TLE1/ZEB1 shRNA clonal lines, the control GFP and GFP-TLE1 A549 cells were transduced with the control shRNA or ZEB1 shRNA lentiviral construct (Santa Cruz Biotechnology, Santa Cruz, CA). 48 hours post-transduction, cells were cultured in the 2 μg/ml puromycin and 20μg/ml blasticidin S (Invitrogen, Carlsbad, CA) to select for positive shRNA expressing clones. Several blasticidin S and puromycin resistant control GFP/control shRNA, GFP-TLE1/control shRNA, and GFP-TLE1/ZEB1 shRNA clones were harvested by cloning rings, and the level of ZEB1 knockdown was confirmed by immunoblotting with a specific ZEB1 antibody. Several control GFP/control shRNA, GFP-TLE1/control shRNA, and GFP-TLE1/ZEB1 shRNA clones were pooled together to generate the control GFP/control shRNA, GFP-TLE1/control shRNA, and GFP-TLE1/ZEB1 shRNA pools, respectively. The stable A549 control shRNA and ZEB1 shRNA pool of cells were generated by transducing the A549 cell line with the lentiviral ZEB1 shRNA or the control shRNA construct (Santa Cruz Biotechnology, Santa Cruz, CA) and cultured in 2 μg/ml puromycin (Invitrogen, Carlsbad, CA). Several puromycin resistant clones were isolated by cloning rings, and the level of ZEB1 knockdown was confirmed by immunoblotting with a specific ZEB1 antibody. Control shRNA and ZEB1 shRNA clones were pooled together to generate the control shRNA and ZEB1 shRNA pools, respectively. The stable control and Bit1mito as well as control shRNA and Bit1shRNA pool of A549 cells were generated as described previously [18, 19].

### Chemical reagents, antibodies, and plasmids

The mouse monoclonal anti-Myc, anti-GFP, and anti-B-actin antibodies were purchased from Sigma (St. Louis, MO). The mouse polyclonal anti-TLE1 and the anti-HDAC1 antibodies were obtained from Abcam (Cambridge, MA). The anti-E-cadherin antibodies Cat #610181 and Cat #610404 were purchased from BD Transduction Laboratories (Lexington, KY). The Polyhema was purchased from Sigma (St. Louis, MO) while blasticin S and puromycin chemicals were obtained from Invitrogen (Carlsbad, CA). The full-length E-cadherin and Myc-tagged ZEB1 plasmid constructs and their corresponding empty vectors were purchased from Origene (Rockville, MD, USA).

### siRNA transfection

The E-cadherin specific siRNA pool, ZEB1 siRNA pool, and the TLE1 specific siRNA pool were purchased from Invitrogen (Carlsbad, CA) [12]. The control, non-targeting siRNA pool was also obtained from Invitrogen (Carlsbad, CA). For transient transfection experiments, cells (2 × 10^5^) were transfected with 25 μM of each siRNA pool by using the Lipofectamine RNAiMAX transfection reagent (Invitrogen, Carlsbad, CA). 48 hrs post-transfection, cells were harvested and subjected to immunoblotting, anoikis, real-time PCR, or reporter assays as described below.

### Cell proliferation, anoikis, and soft agar assays

As described previously [12], anchorage-dependent growth was determined by plating cells in a volume of 100 μl at a density of 6,000 cells per well in 96-well plates. At each indicated time, the number of metabolically active cells was measured by alarmar blue staining (Invitrogen) and fluorescence reading at 485 nm excitation wavelength and 520 nm emission wavelength with a microplate plate reader. To assess for anoikis cell death, cells were grown in suspension by plating cell onto Polyhema coated 96 well plates in complete growth medium containing 0.5% methylcellulose at a density of 1.0 × 10^4^/well as previously described [12]. Following 30h for A549 cells and 24h for BEAS-2B cells, detached cells were then collected and subjected to the Cell Death ELISA apoptosis assay (Roche Molecular Biochemicals, Indianapolis, IN) which quantitates the level of cytosolic nucleosomal fragments [12]. In parallel, cells grown in normal cell culture conditions (attached cells) were also subjected to Cell Death ELISA apoptosis assay. Anchorage-independent growth was determined by culturing cells in soft agar using the 96-well plate format. As described previously [12], 5,000 cells in 0.3% agar solution was plated onto wells precoated with 0.6% agar in the culture medium. The growth of the resulting colonies was quantified by counting the number of visible colonies (with a diameter greater than 30 uM) and by alamar fluorescence assay as described previously [12].

### Protein preparation and immunoblotting assays

Total cell lysate preparation and Western blotting were performed as described previously [12]. Briefly, cells were harvested by adding ice-cold NP-40 lysis buffer (1% NP-40; 20mM Tris-HCL[pH7.4]; 150 mM NaCl; 10% glycerol, 2 mM sodium vanadate; 1 mM phenylmethylsulfonyl fluoride; 10 μg/ml leupeptin; and 5 μg/ml aprotinin) and incubated at 4°C for 20 min followed by centrifugation at 14,000 rpm to remove insoluble debris. Plasma membrane protein lysate was prepared using the Mem-PER Plus eukaryotic membrane protein extraction reagent kit (Pierce) as described previously [33]. For immunoblot analysis, equal amounts of total cell and membrane protein lysate were resolved on 4-20% gradient Tris-glycine gels (Invitrogen) and electrophoretically transferred to nitrocellulose membrane. The membranes were incubated with primary antibodies overnight at 4°C followed by secondary antibodies conjugated with horseradish peroxidase. Membranes were developed using the ECL detection system.

### Coimmunoprecipitation assay

Coimmunoprecipitation assay was performed as described previously [15]. Briefly, A549 cells were resuspended in ice-cold Nonidet P-40 lysis buffer (1% Nonidet P-40, 20 mm Tris-HCl, pH 7.4, 150 mm NaCl, 10% glycerol, 2 mm sodium vanadate, 1 mm phenylmethylsulfonyl fluoride, 10 μg/ml leupeptin, and 5 μg/ml aprotinin) followed by a 20-min incubation at 4°C. Cell debris was removed by centrifugation. The resulting lysate was immunoprecipitated with anti-TLE1 (C7, Santa Cruz Biotechnology, Santa Cruz, CA), anti-ZEB1 (H-102, Santa Cruz Biotechnology, Santa Cruz, CA), or non-specific IgG (3H1190, Santa Cruz Biotechnology, Santa Cruz, CA) and thoroughly washed with lysis buffer. Bound proteins were resolved by SDS-PAGE, and immunoblotting was performed using anti-TLE1 (ab155046, Abcam, Cambridge, MA) or anti-ZEB1 (ABE596, EMD Millipore, Billerica, MA) antibody. In other experiments, control GFP and GFP-TLE1 A549 cells were transfected with the Myc-ZEB1 or empty vector construct and harvested by washing once with PBS and resuspended in ice-cold Nonidet P-40 lysis buffer followed by a 20-min incubation at 4°C. Cell debris was removed by centrifugation. GFP-tagged TLE1 was immunoprecipitated with anti-GFP-agarose conjugate (Abcam, Cambridge, MA) and thoroughly washed with lysis buffer. Bound proteins were resolved by SDS-PAGE, and Western blotting was performed using anti-Myc antibody.

### Total RNA extraction and quantitative real-time PCR

As described previously [15], total RNA was extracted from 1.0 × 10^6^ cells using the RNeasy kit (Qiagen) and quantified by spectrophotometry (NanoDrop 8000, Thermo Scientific). In certain experiments, cells were grown in suspension by culturing cells onto poly-hema coated wells for 2h (A549) and 3h (BEAS-2B) prior to RNA extraction. Total RNA was subjected to a one-step real-time RT-PCR using the iTaq Universal SYBR Green One-Step Kit (Bio-Rad) and cDNA quantification by real-time PCR on the BIO-RAD iQ5 Multicolor Real-Time PCR Detection System using the human E-cadherin (forward primer: AGGCTAGAGGGTCACCGCGTC and reverse primer: GCTTTGCAGTTCCGACGCCAC). The human GAPDH primers (forward primer: CCCACTCCTCCACCTTTGAC and reverse primer: TTGCTGTAGCCAAATTCGTTGT) were used for control. The Real-time PCR experiments were performed at least three times.

### Promoter luciferase analysis

The cell-based reporter luciferase assay was performed as described previously [15]. Briefly, A549 and BEAS-2B cells were cotransfected with the E-cadherin luciferase promoter-reporter construct (SwitchGear Genomics) and the GAPDH luciferase promoter-reporter vector (SwitchGear Genomics) which serves as an internal control for luciferase activity. Following 24 h incubation, cells were grown in suspension for 12h for A549 and 7h for BEAS-2B and then harvested and subjected to a luciferase assay (SwitchGear Genomics’ LightSwitch Luciferase Assay System) following the manufacturer's protocol. Luciferase activity was normalized to GAPDH luciferase activity and the relative luciferase activity was presented. To determine the effect of ZEB1 knockdown on E-cadherin promoter activity in TLE1 expressing cells, control GFP/control shRNA, GFP-TLE1/control shRNA, and GFP-TLE1/ZEB1 shRNA A549 derived cells were cotransfected with the E-cadherin luciferase promoter-reporter construct (SwitchGear Genomics) and the GAPDH luciferase promoter-reporter vector (SwitchGear Genomics). Following 24h incubation, the cells were subjected to a luciferase assay as described above. The promoter luciferase experiment was performed at least three times.

### ChIP assay

As described previously [15], cells grown to 70% to 80% confluence were crosslinked with 1% formaldehyde and processed using the EpiSeeker ChIP Kit – One Step (Abcam). The resulting chromatin fragments were immunoprecipitated with the anti-TLE1 antibody-ChIP Grade (Abcam), anti-Acetyl-Histone H3-ChIP grade (anti-Ac-H3) (EMD MILLIPORE), or non-specific IgG (Santa Cruz Biotechnology). Subsequent downstream steps were conducted following the protocol from the EpiSeeker ChIP Kit (Abcam). The PCRs were conducted using the E-cadherin primers 5′ GAC CGA GAG AGT TTC CCT ACG 3′ (forward) and 5′ TCA GGC ACC TGA CCC TTG TA 3′ (reverse) with the following program: 45 cycles at 95°C for 30 s, 56°C for 30s, and 72°C for 30s. The amplified 158 bp E-cadherin DNA promoter (−1000 to −1158) fragment was separated on 1.5% agarose gel and visualized with ethidium bromide [34]. The purified DNA samples were used as an input for PCR reactions. The PCR product was then subjected to densitometric quantification using QuantityOne software (Bio-Rad). Fold enrichment was determined and expressed as a +TLE1-specific antibody, +ZEB1-specific antibody, or +Acetyl-Histone H3-specific antibody over non-specific IgG. The ChIP experiments were repeated at least three times.

### *In vivo* tumorigenesis assay

All procedures were done according to protocols approved by the Institutional Committee for Use and Care of Laboratory Animals of Xavier University of Louisiana Institutional Animal Care and Use Committee (IACUC, Approval Number 060911-001BI). Eight-week-old female athymic nude mice (BALB/c) were used for the tumorigenesis assay [18]. The A549 derived control GFP/control shRNA, GFP-TLE1/control shRNA, and GFP-TLE1/ZEB1 shRNA (1.0 × 10^6^) were injected subcutaneously (5 animals/group), and the tumor sizes were measured periodically with a caliper at the indicated time points. Tumor volume was determined by the formula (d1×d2^2^)/2 where d1 represents the larger diameter and d2 the smaller diameter. Mice were sacrificed when the primary tumors reached 2 cm in diameter and the resulting tumors were harvested and weighed.

### Statistical analysis

Data are presented as means (±S.D.). For western blots and ChIP assays, experiments were performed at least three times. Statistical differences between groups were established at a P value < 0.05 using the Student's t-test (two-tailed). All calculations were done using the NCSS statistical software (NCSS, Kaysville, UT).

## SUPPLEMENTARY FIGURES



## Abbreviations

TLE1: transducin-like enhancer of split 1
EMT: epithelial-to-mesenchymal transition
Bit1: Bcl-2 inhibitor of transcription 1
