# Supplementary material for: TLE1 inhibits anoikis and promotes tumorigenicity in human lung cancer cells through ZEB1-mediated E-cadherin repression
Source: Oncotarget. 2017 Jul 31;8(42):72235–49. doi: 10.18632/oncotarget.19703 (PMC5641126; doi:10.18632/oncotarget.19703)
Supplement: Supplementary file 1 [file oncotarget-08-72235-s001.pdf]

TLE1 inhibits anoikis and promotes tumorigenicity in human lung cancer cells through ZEB1-mediated E-cadherin repression

SUPPLEMENTARY MATERIALS

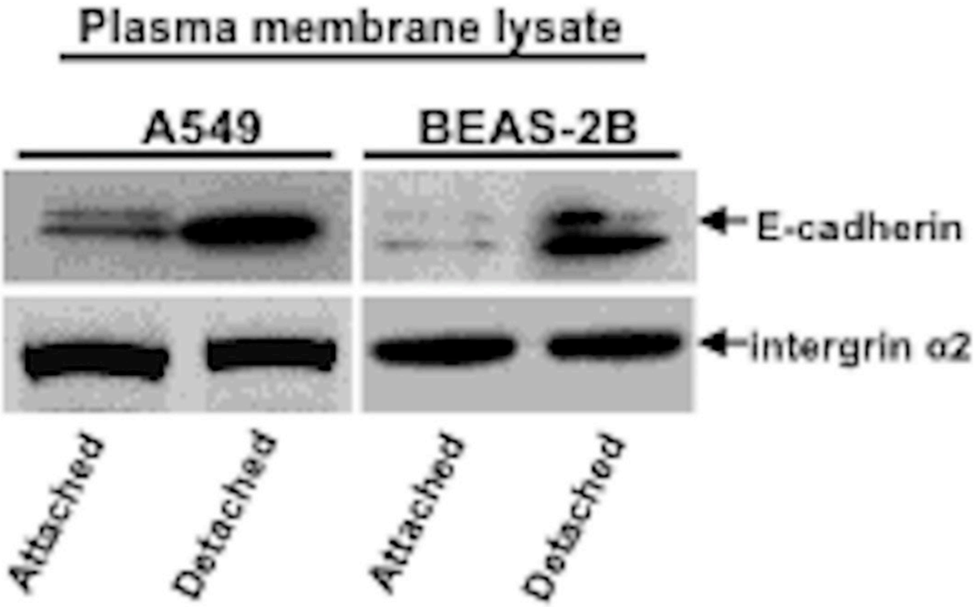

Supplementary Figure 1: Loss of cell attachment increases E-cadherin plasma membrane localization. A549 and BEAS-2B cells were cultured in normal culture condition (attached) or in suspension (detached) as described in Materials and Methods. Cells were then harvested and subjected to plasma membrane protein extraction and the resulting membrane lysate was immunoblotted with the anti-E-cadherin and anti-integrin α2 (plasma membrane marker) antibodies.

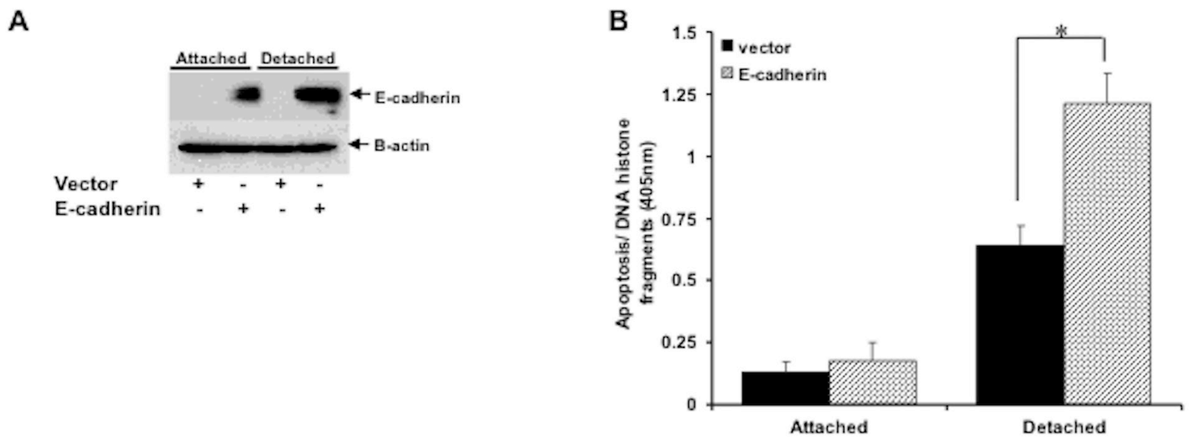

Supplementary Figure 2: Exogenous E-cadherin expression induces anoikis in H460 cells. **(A** and **B)** H460 cells were transfected with the empty vector or E-cadherin construct, and 24h later cells were harvested and cultured in normal culture condition (Attached) or in suspension (Detached) for 24h. Cells were subsequently subjected to immunoblotting with the indicated antibodies (A) and cell death ELISA assay (B). In B, three independent experiments were performed in triplicates, * indicates $p<0.05$ by Student's t test.

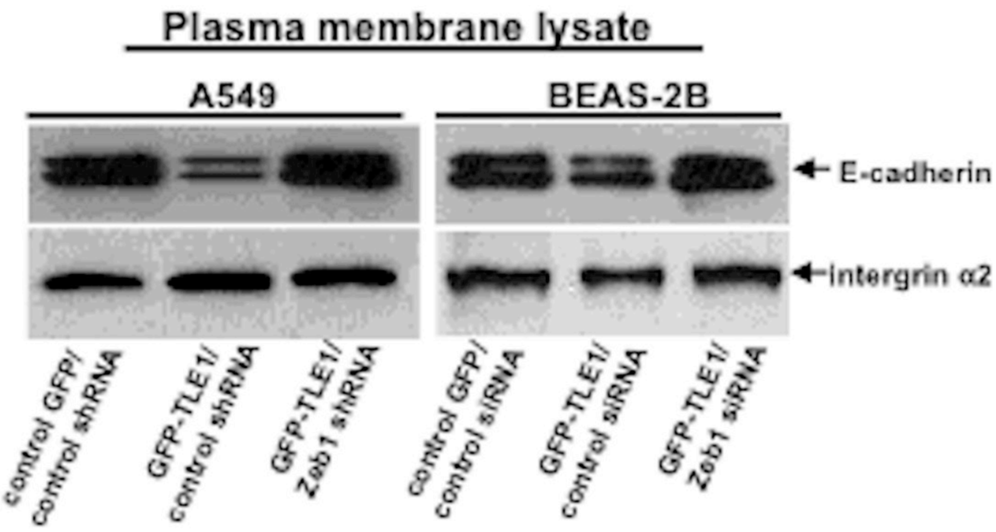

Supplementary Figure 3: The TLE1-mediated decrease in E-cadherin plasma membrane localization is attenuated by Zeb1. Control GFP/control shRNA, GFP-TLE1/control shRNA, and GFP-TLE1/ZEB1 shRNA A549 cells and control GFP and GFP-TLE1 BEAS-2B cells treated with control or ZEB1 siRNAs were subjected to plasma membrane protein extraction and the resulting plasma membrane lysate was immunoblotted with the anti-E-cadherin and anti-integrin α2 (plasma membrane marker) antibodies.

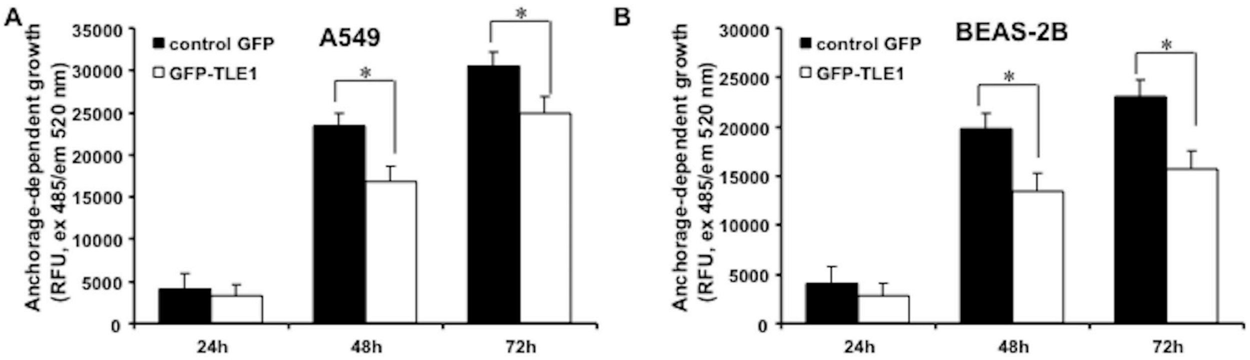

Supplementary Figure 4: Exogenous TLE1 expression inhibits the anchorage-dependent growth of A549 and BEAS-2B cells. **(A** and **B)** Stable control and TLE1 expressing pool of A549 (A) and BEAS-2B (B) cells were plated onto regular tissue culture plates and the growth of cells was quantified by alamar fluorescence assay at the indicated time points. In A and B, three independent experiments were performed in triplicates, * indicates $p<0.05$ by Student's t test.
